# Synergistic Effect of MWCNT and CB on the Piezoresistive Properties of Laser Ablation Composites Strain Sensors

**DOI:** 10.3390/nano15130997

**Published:** 2025-06-26

**Authors:** Shikang Yin, Richao Tan, Sitian Wang, Yuan Yuan, Kaiyan Huang, Ziying Wang, Shijie Zhang, Sadaf Bashir Khan, Weifeng Yuan, Ning Hu

**Affiliations:** 1 Key Laboratory of Testing Technology for Manufacturing Process, Ministry of Education, School of Manufacturing Science and Engineering, Southwest University of Science and Technology, Mianyang 621010, China; yinshikang@mails.swust.edu.cn (S.Y.); sitianwang@mails.swust.edu.cn (S.W.); swustyy@mails.swust.edu.cn (Y.Y.); sadafbashirkhan@swust.edu.cn (S.B.K.); yuanweifeng@swust.edu.cn (W.Y.); 2 College of Mechanical and Vehicle Engineering, Chongqing University, Chongqing 400044, China; tanxuanqing2003@163.com; 3 College of Information Technology, Luoyang Normal University, Luoyang 471934, China; zhangshijie@lynu.edu.cn; 4 Interdisciplinary Research Institute of Advanced Intelligent Equipment, Xihua University, Chengdu 610039, China; ninghu@hebut.edu.cn

**Keywords:** MWCNT, CB, PDMS, laser ablation, strain sensor

## Abstract

A flexible and highly sensitive piezoresistive strain sensor was fabricated through the application of CO_2_ laser ablation on a composite film composed of multi-walled carbon nanotubes, carbon black, and polydimethylsiloxane (MWCNT/CB/PDMS). The results of scanning electron microscopy (SEM) surface analysis shows that the “bush-like” conductive structure on the PDMS-based composite material membrane post-laser ablation is formed. Transmission electron microscopy (TEM) images and X-ray diffraction (XRD) spectra of the ablation products indicated the formation of an amorphous carbon layer on the surface of carbon nanomaterials due to laser ablation. Experimental findings revealed that the sensitivity (GF) value of the sensor based on CNT0.6CB1.0-P3.0 is up to 584.7 at 5% strain, which is approximately 14% higher than the sensitivity 513 of the sensor previously prepared by the author using CO_2_ laser ablation of MWCNT/PDMS composite films. The addition of a very small volume fraction of CB particles significantly enhances the piezoresistive sensitivity of the sensor samples. Combined with the qualitative analysis of microscopic morphology characterization, CB and MWCNT synergistically promote the deposition of amorphous carbon. This phenomenon increases the probability of tunnel effect occurrence in the strain response region of the sensor, which indirectly confirms the synergistic enhancement effect of the combined action of CB and MWCNT on the piezoresistive sensitivity of the sensor.

## 1. Introduction

Composites, including polymers and carbon nanofillers have garnered significant interest because of their improved mechanical, thermal, and electrical characteristics. The piezoresistive properties of a homogeneous mixture of conductive composites doped with single carbon nanomaterials have been systematically and well studied in academia [1,2,3,4]. The variation in the external dimensions of the materials on the macroscopic scale, the formation and disconnection of the conductive network on the microscopic scale, and the quantum tunneling effect of the electrons on the nanoscopic scale collectively form the theoretical basis for the piezoresistive effect of the carbon nanoparticles/polymers composites [5]. The synergistic effects of hybrid filler systems (two or three nanofillers) on mechanical and electrical properties of nanocomposites have recently been demonstrated [6,7,8,9,10,11,12,13]. Ismail et al. and Yan et al. found that the addition of MWCNT and CB synergistically enhanced the mechanical properties of rubber composites [14,15]. Nakaramontri et al. demonstrated a significant improvement in the electrical conductivity of the nanocomposites by using conductive carbon black to fill the gaps between CNT encapsulations in natural rubber composites [16]. Wu et al. reported that multi-walled carbon nanotubes and graphene oxide can synergistically enhance the mechanical and separation properties of polyelectrolyte composite membranes [17]. Huang et al. found that CB/MWCNT/polyvinylidene fluoride hexafluoropropylene (PVDF-HFP) composite films were fabricated as strain sensors at a given loading well below the percolation threshold of a single filler, and mixed nanofillers could effectively improve the conductivity and sensitivity of the nanocomposites [18].

Compared with the traditional metal foil strain gauges, carbon nanofiller/polymer composite sensors have the advantages of high sensitivity and strain range. However, due to the limitation of the traditional homogeneous mixing process, the carbon nanofillers can only be homogeneously mixed in a certain proportion in the polymer matrix to form a macroscopically homogeneous microscopic conductive network, which limits the further enhancement of the composite’s piezoresistive response characteristics in terms of sensitivity, linearity, and stability. To further improve the sensing performance of carbon nanocomposite sensing components, some special processing processes have been introduced into the research and development of composite sensing components, such as the chemical modification process for nanofillers [19], electromagnetic field-assisted process for nanofillers [20], 2D spraying process for thin-film nanocomposites [21,22,23], 3D printing process spatially structured nanocomposites [24,25], and the freeze-drying process for porous nanocomposites [26,27]. Compared with the homogeneous mixing process, the spraying process is more suitable for fabricating thin-film composite sensors. By adjusting the spraying thickness and speed, thin-film strain sensors can be developed quickly and effectively [21,22,23]. The 3D printing process has an inherent advantage in creating artificially controllable macroscopic conductive pathways in space. It can effectively regulate the performance of nanocomposite sensors from two aspects: the micro-functional modification of functional inks and the design of 3D printing paths [24,25]. The freeze-drying process is widely used in the preparation of porous nanocomposites [26,27]. By controlling parameters such as quenching time and vacuum time, the porosity of the composites can be regulated, making it widely applicable to the fabrication of pressure sensors. Direct laser ablation has become a popular means of developing high-performance functional composites today due to its convenient and flexible processing characteristics and the accompanying high-temperature reaction environment. The laser ablation process can be categorized into two main groups based on the role of the laser in the composite sensing components.

**Figure 1:**
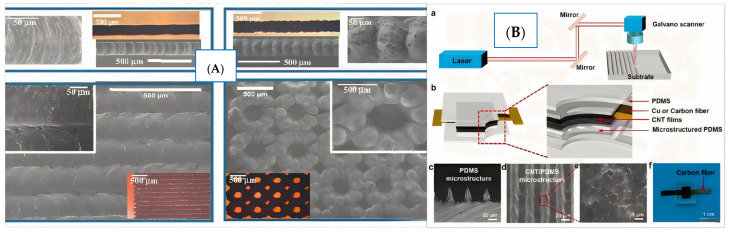
(**A**) SEM and optical images of laser-induced graphite structure with various patterns [35]; (**B**) schematic diagram of laser microengineering of composite sensor [37]: (**a**) A schematic showing the laser microengineering of PDMS; (**b**) A schematic exhibiting the configuration of the pressure sensor; (**c**) An optical image of a laser microengineered PDMS; (**d**,**e**) SEM images showing a laser-microengineered electrode; (**f**) A photograph of a laser-microengineered device.

The initial category involves the laser as a reaction condition, where the concentrated energy produces elevated temperatures in certain areas. The high temperature induces chemical reactions, such as the reduction in graphene oxide (GO), inside the region exposed to the laser [28,29,30,31,32] and the carbonization of polymer matrix [33], etc., which, in conjunction with the direct laser writing (DLW) method, allows for the ablation of a special texture according to the expected properties. Tian et al. proposed a simple one-step laser direct writing method to prepare reduced graphene oxide (rGO) [29,30]. The stacked rGO layers are used as the sensing structure of strain sensors, which can be designed according to the needs. These strain sensors have good piezoresistive response and operational stability. Zhu et al. used the DLW method to obtain the rGO. They used it to prepare a composite pressure sensor based on an asymmetric composite pressure sensor with a bilayer structure [32]. This piezoresistive pressure sensor is suitable for creating electronic skin due to its high sensitivity and rapid reaction time. In addition, this composite sensor can detect finger touch and pulse in real time. Gong et al. prepared a high-sensitivity flexible strain sensor based on rGO/PS by using DLW method in conjunction with a process of polystyrene (PS) nanoparticle doping [34]. PS nanoparticles acted as insulators, and separated and intercalated stacked rGO lamellae, thus constructing a partially connected sparse conductive network. Therefore, modifying PS nanoparticles can significantly improve the electrical properties of rGO films. Liu et al. reported a method for preparing porous graphite structures based on DLW [33,35], as shown in Figure 1A. By laser ablation, polyimide (PI) was transformed into a porous graphite structure. This process was used to prepare flexible piezoresistive sensors and sensor arrays on PI films. The performance of the sensors could be optimized by adjusting the laser ablation pattern and the magnitude of the power-to-velocity ratio. Milenov T et al. put pulsed laser ablation (PLA) into microcrystalline graphite suspensions in an aqueous medium to obtain the fine suspension-like graphene phases (such as defective graphene, graphene oxide, and reduced graphene oxide) [36]. In the second category, the laser acts as a “cutter” in the processing. The high-energy laser beam can cut and shape, and by removing the excess material, microstructures with certain special properties can be fabricated. Lu et al. reported a carbon nanotube (CNT)/polydimethylsiloxane (PDMS) pressure sensor, microfabricated by laser microfabrication engineering, which exhibited 10 times higher sensing sensitivity compared to the test sample without microfabrication [37], as shown in Figure 1B.

Herein, we utilize a hybrid filler system and combine the characteristics of two laser ablation processes. We employ a CO_2_ laser (wavelength: 10,600 nm) to ablate PDMS-based composites containing MWCNT and CB, ensuring their contents are below the percolation threshold. This approach allows us to create a conductive structure resembling a “bush” with a high sensitivity to piezoresistive response. Building upon this, we develop and fabricate flexible strain sensors using MWCNT/CB/PDMS.

## 2. Experiment

### 2.1. Materials

In this paper, MWCNT is selected from NT-7K type products produced by NEC Corporation, Tokyo, Japan, with an average diameter of 50 nm, length of 0.5 to 1 μm, and purity higher than 99.5% (purity measured by tungsten filament X-ray); CB is selected from Vulcan XC-72 type products produced by Cabot Corporation, Boston, MA, USA; PDMS is selected from DC184 type products produced by Dow Corning, Midland, MI, USA; the coating machine is selected from TB-500 type products produced by Chongqing Tongxi Science and Technology, Chongqing, China. According to the experimental planning, the composites prepared with different types and amounts of carbon nanomaterials are summarized in Table 1.

**Figure 2:**
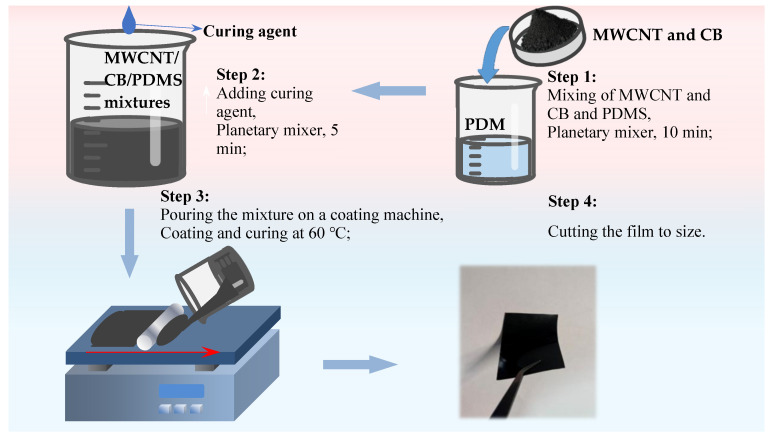
Fabrication process of the MWCNT/CB/PDMS nanocomposite film.

The specific steps for the preparation of the composite films are as follows, as shown in Figure 2:

① According to the experimental planning, MWCNT and CB were placed in PDMS with planetary stirring at 1000 r/min for 10 min.

② The composite molding precursor was prepared by adding an appropriate amount of curing agent to the mixture obtained in step 1 and planetary stirring at 1000 r/min for 5 min.

③ The viscous liquid precursor of carbon nanofiller/PDMS composites was dumped on the coating machine and cured at 60 °C for 3 h after the coating was completed.

④ The cured MWCNT/CB/PDMS composite film is carefully removed from the coater release paper and cut to shape as needed.

### 2.2. Fabrication of the Composite Strain Sensor with Laser Ablation

**Figure 3:**
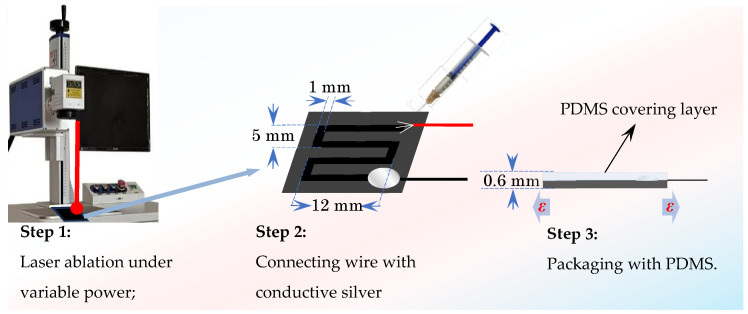
Fabrication process of composite sensors with laser ablation.

The prepared MWCNT/CB/PDMS composite film was cut into a rectangular film of 20 mm × 18 mm and laser-ablated to prepare the MWCNT/CB/PDMS laser-ablated strain sensors, as shown in Figure 3:

① The cut rectangular composite film was laid flat on the laser ablation liner to expel air bubbles.

② The paths and dimensions were ablated using lasers of different powers at 30 mm/s, as shown in Figure 3.

③ Conductive silver adhesive to solder the wire or electrode to the end of the ablation pattern was used.

④ Overlay encapsulation protection of ablation patterns using pure PDMS.

Using the material sample numbers provided in Table 1 and varying laser ablation powers, the composite strain sensor test samples were assigned numbers as shown in Table 2.

### 2.3. Experimental Tests

We found that carbon nanofillers with different aspect ratios produce a synergistic enhancement effect in the polymer matrix [18]. In order to explore whether this synergistic effect also occurs in composite sensors prepared by the laser ablation process, experimental tests are designed to evaluate the piezoresistive performance of laser-ablated strain sensors of MWCNT/CB/PDMS composites. The conductivity of the effective working area of the sensor is a crucial parameter. When the change in resistance remains constant, a lower initial resistance can yield higher piezoresistive sensitivity. Generally, the four-probe testing method is employed to measure the conductivity of macroscopic homogeneous materials. However, in this study, the conductive layer generated by laser ablation is relatively fluffy. The four-probe testing method would damage its conductive structure. Therefore, a high-resolution digital multimeter (Model 34465A, Agilent Technologies, Santa Clara, CA, USA) was utilized to directly measure the initial resistance of the sensor samples, providing an approximation for evaluating the conductivity of the laser-ablated structures. The initial resistances of the sensor samples are presented in Table 3. It should be noted that since the maximum resistance range of the high-precision multimeter is 100 MΩ, the initial resistances of some samples exceeding this range are not reported.

By analyzing the data in Table 3, we can obtain the effect of adding a small amount of CB on the initial resistance of the film sheet, as shown in Table 4. Under different CNT ratios, the effect of CB on the conductivity of the film sheet may be gain or degrade.

## 3. Results and Discussion

### 3.1. Sensing Performance of MWCNT/CB/PDMS Composite Laser-Ablated Strain Sensors

**Figure 4:**
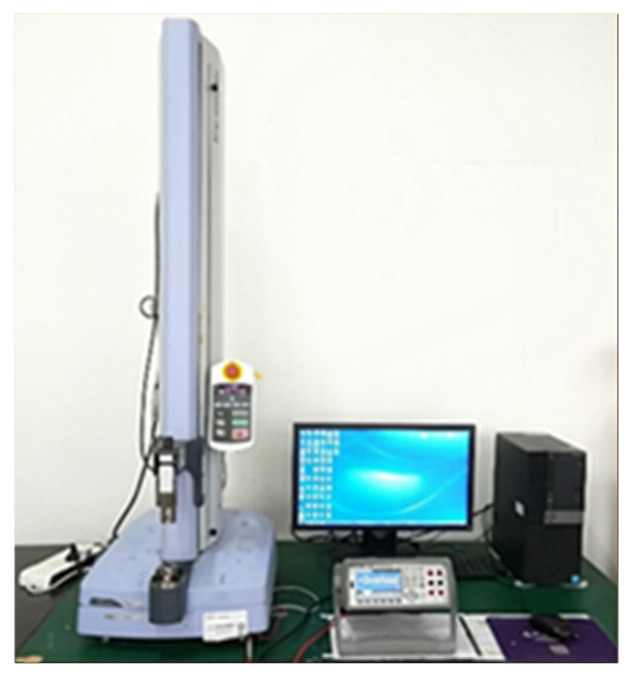
The piezoresistive performance test system of the composite strain sensor.

To assess the impact of MWCNT and CB content, as well as laser power, on the piezoresistive sensing characteristics of laser-ablated MWCNT/CB/PDMS composite sensors, the testing method depicted in Figure 4 was employed.

**Figure 5:**
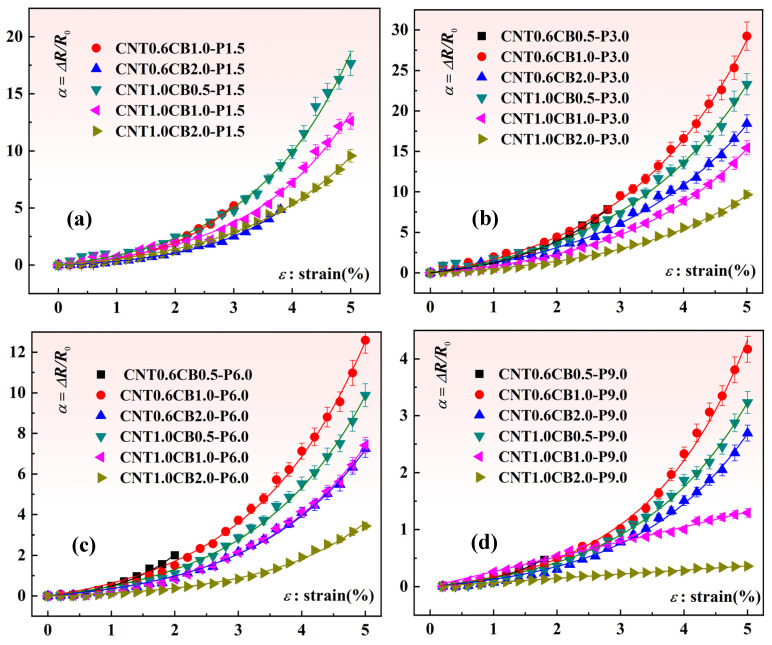
Effect of the CNT and CB addition and the laser power on the piezoresistivity of the composite sample: (**a**) Effect of CNT and CB loading on the piezoresistivity of composite specimens (Laser Power: 1.5 W); (**b**) Effect of CNT and CB loading on the piezoresistivity of composite specimens (Laser Power: 3.0 W); (**c**) Effect of CNT and CB loading on the piezoresistivity of composite specimens (Laser Power: 6.0 W); (**d**) Effect of CNT and CB loading on the piezoresistivity of composite specimens (Laser Power: 9.0 W).

**Figure 6:**
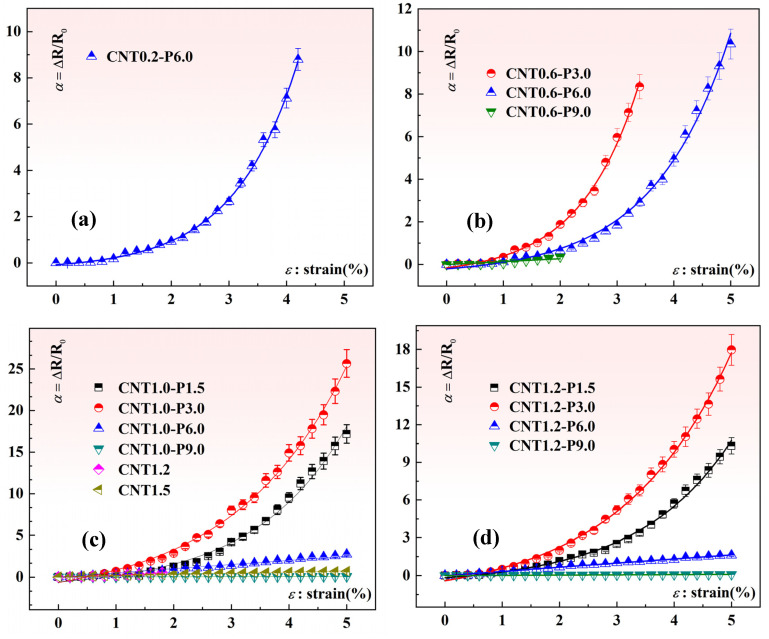
Effect of the CNT addition and the laser power on the piezoresistivity of the composite sample [38]: (**a**) Effect of laser power on the piezoresistivity of composite specimens (with 0.2 wt.%CNT); (**b**) Effect of laser power on the piezoresistivity of composite specimens (with 0.6 wt.%CNT); (**c**) Effect of laser power on the piezoresistivity of composite specimens (with 1.0 wt.%CNT); (**d**) Effect of laser power on the piezoresistivity of composite specimens (with 1.2 wt.%CNT).

Specifically, quasi-static mechanical tensile experiments were conducted to evaluate the piezoresistive performance of the composite material strain sensors. The procedure involved securing the laser-ablated MWCNT/CB/PDMS composite material sensors using pneumatic membrane fixtures within a uniaxial tensile testing machine (EZ-LX, Shimadzu, Japan). During the mechanical loading process, the resistance variations in the composite material strain sensors were measured using the high-resolution digital multimeter. The relevant digital signals were synchronized in real-time through transmission cables in computer acquisition software. The relationship between the strain and resistance variations in the composite material samples was analyzed. In this study, three replicates of each sample category were prepared, and three experiments were conducted for each sample. Figure 5 illustrates the piezoresistive properties curves of the composite material sensor test samples prepared under different laser power ablation conditions with varying amounts of MWCNT and CB additives. In contrast, Figure 6 presents the experimental results of the piezoresistive performance of our previously fabricated laser ablation strain sensors with only MWCNT added [38]. It is noteworthy that some data in the figure are incomplete. This is because during the loading process, the resistance signals of the sensor samples exceeded the maximum measurement range of the measuring instrument. We believe that the effective conductive network has been completely disrupted at this point.

**Figure 7:**
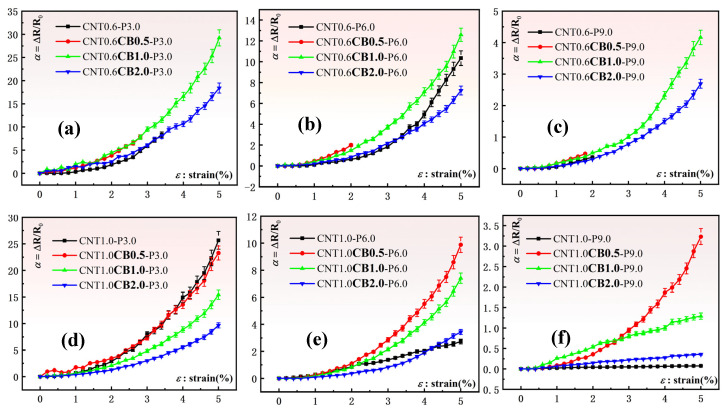
The influence of different mass fractions of CB on the resistivity of sensors (under the same CNT content and laser power): (**a**) Under 0.6 wt.%CNT and 3.0 W laser power; (**b**) Under 0.6 wt.%CNT and 6.0 W laser power; (**c**) Under 0.6 wt.%CNT and 9.0 W laser power; (**d**) Under 1.0 wt.%CNT and 3.0 W laser power; (**e**) Under1.0 wt.%CNT and 6.0 W laser power; (**f**) Under 1.0 wt.%CNT and 9.0 W laser power.

Through rigorous data analysis, we compared the resistance change rates of CB thin-film sheets with different mass fractions added under the same CNT content and laser power to clarify the effect of CB on the sensing performance of the thin-film sheets, as shown in Figure 7.

**Figure 8:**
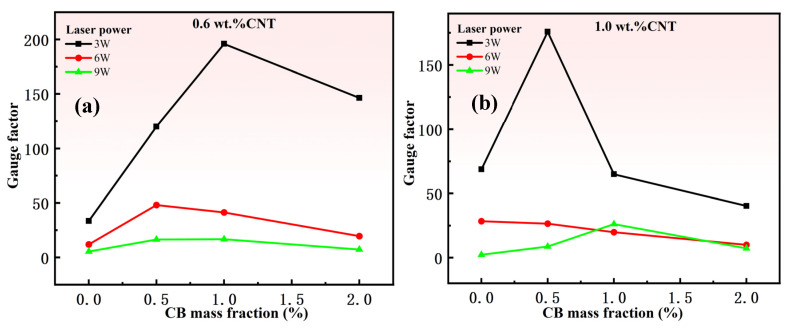
(**a**) Effect of different mass fractions of CB on sensor sensitivity at 1% strain (0.6 wt.% MWCNT content and same laser power). (**b**) Effect of different mass fractions of CB on sensor sensitivity at 1% strain (1.0 wt.%CNT content and same laser power).

From Figure 7a–c, we can see that with the addition of 0.6 wt.%CNT and the same laser ablation power, the sensitivity of the thin-film sheet sensor with the addition of 1.0 wt.%CB (marked by the green curve in the figure) is better than that with only the addition of CNT (marked by the black curve in the figure), and it can also be seen from the figure that the thin-film sheet sensor with only CNT added is unable to measure the resistance change rate when the strain reaches about 5%, at which point the sensor fails. However, the thin-film sheet sensor with 1.0 wt.%CB added can maintain good sensing performance at a strain of 5%. As in Figure 7d–f, when adding 1.0 wt.%CNT and with the same laser ablation power, the sensitivity of the thin-film sheet sensor with 0.5 wt.%CB added (marked by the red curve in the figure) is better than that with only CNT added (marked by the black curve in the figure). This indicates that under appropriate laser power and CNT content, adding a trace amount of CB can indeed effectively improve the sensing performance of the thin-film sheet. Then to ensure the integrity of the sensitivity data, we choose the sensitivity at 1% strain, as shown in Figure 8.

As can be seen from Figure 8, with the increase in CB, the sensitivity of the sensor shows a trend of first increase and then decrease. It can be confirmed from the figure that under the same CNT content and laser power, adding a small amount of CB can indeed effectively improve the sensitivity of the sensor.

**Figure 9:**
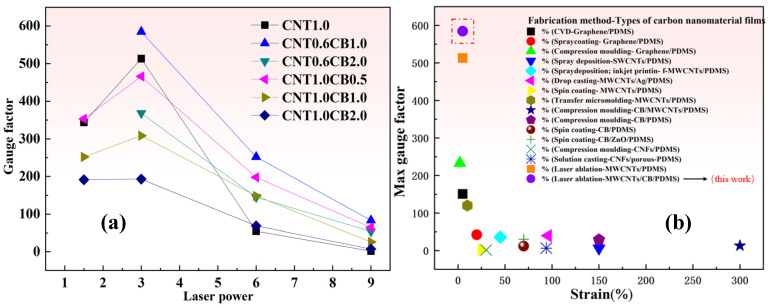
(**a**) The effect of MWCNT and CB content and laser power on the gauge factor of the composite samples at the 5% strain. (**b**) Comparison of the sensitivity of PDMS-based using different types of carbon nanoparticles and different preparation methods [1].

To assess the sensing stability of laser-ablated MWCNT/CB/PDMS composite material strain sensors, sample CNT0.6CB1.0-P3.0, exhibiting the best sensitivity at 5% strain, as depicted in Figure 9, was selected. When compared with the sample CNT1.0-P3.0, the sensitivity of the sample CNT0.6CB1.0-P3.0 at a strain of 5% was increased from 513 to 584.7, representing an approximate 14% improvement. In addition, compared with the previous studies of scholars, in the flexible sensors fabricated based on PDMS, the flexible sensors fabricated by laser ablation in this paper exhibit higher sensitivity at the same level of flexibility [1].

**Figure 10:**
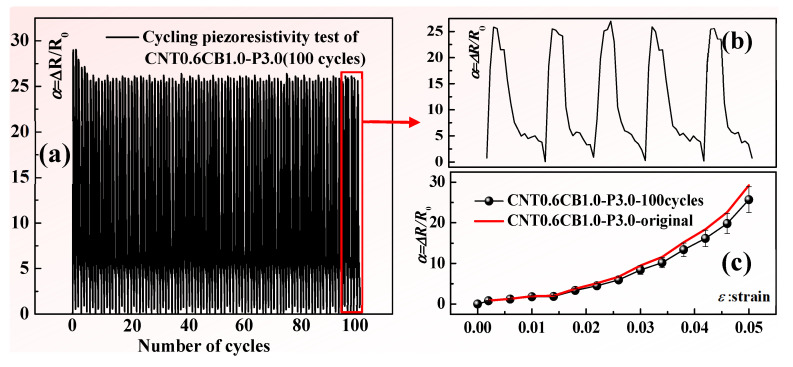
(**a**) Hundred-cycle piezoresistive durability test of sample CNT0.6CB1.0-P3.0 at 5% strain; (**b**) amplified representation of five representative cyclic responses from (**a**); (**c**) single-cycle piezoresistive performance test results after hundred-cycle durability experiment.

Employing the testing methodology illustrated in Figure 4, the relationship between strain and resistance variations in the composite material samples was examined. Tensile testing was conducted with a strain rate set at 5 mm/min and a strain range of 0 to 0.05, with 100 cycles performed to assess the cyclic durability of sample CNT0.6CB1.0-P3.0. Figure 10a presents the piezoresistive response of sample CNT0.6CB1.0-P3.0 during 100 cycles of loading experiments, demonstrating a minor performance decay at the beginning of the cyclic loading, followed by relatively stable results. Figure 10b displays the cyclic results of five responses, showing no significant decay, fluctuation, or noise in the experimental result. Subsequently, the samples subjected to 100 cycles of fatigue testing were subjected to single-cycle tensile testing three times, and the average values were calculated along with their standard deviations, as depicted in Figure 10c. The results indicate that after experiencing 100 cycles of fatigue testing, sample CNT0.6CB1.0-P3.0 strain sensor retains a relatively stable piezoresistive response, with only minor performance decay compared to the initial results.

### 3.2. Microstructure Characterization and Mechanism Analysis

**Figure 11:**
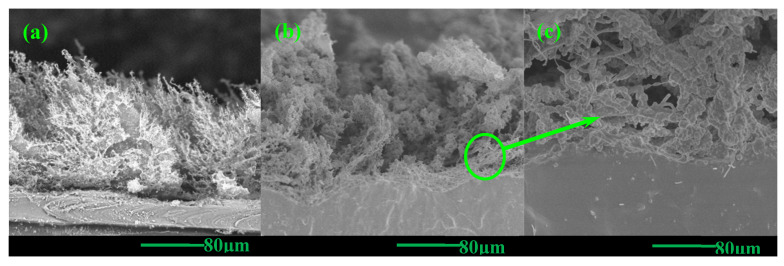
(**a**) Cross-sectional morphology of CNT1.0-P3.0 [38]; (**b**) cross-sectional morphology of CNT0.6CB1.0-P3.0; (**c**) cross-sectional morphology details of CNT0.6CB1.0-P3.0.

In order to investigate the synergistic effect of MWCNT and CB in laser-ablated composite material strain sensors, microscopic characterization of the laser-ablated region of MWCNT/CB/PDMS composite materials is essential. Similarly, low-temperature fracturing of sample CNT0.6CB1.0-P3.0 in a liquid nitrogen environment was performed to obtain flat-fracture surfaces, followed by observation of the cross-sectional microstructure of the laser-ablated samples using scanning electron microscopy (7610FSEM, JEOL Ltd., Tokyo, Japan). As depicted in Figure 11, panels (a) and (b) illustrate the cross-sectional microstructures of samples CNT1.0P3.0 and CNT0.6CB1.0-P3.0, respectively. It is observed that sample CNT0.6CB1.0-P3.0 exhibits a laser-ablated structure similar to that of sample CNT1.0P3.0, resembling a “bush-like” morphology. However, upon closer examination of the cross-sectional microstructure of sample CNT0.6CB1.0-P3.0, as shown in the locally enlarged view in Figure 11c, MWCNT serve as the structural backbone of the ablated structure. At the same time, some granular material is embedded within the externally coated amorphous carbon, presumed to be CB particles encapsulated in amorphous carbon.

**Figure 12:**
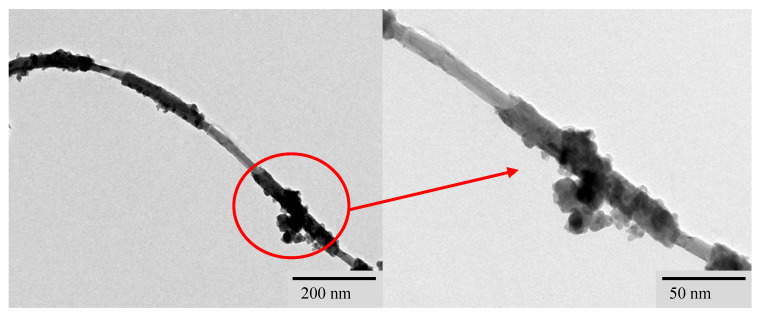
TEM images of the boscage-like structure of sample-CNT0.6CB1.0-P3.0 in high resolution.

To further characterize the ablation products of sample CNT0.6CB1.0-P3.0, the ablation products on the sample’s surface were scraped off using a surgical blade after laser ablation. The ablation product powder was then subjected to ultrasonication in anhydrous ethanol for 30 s, followed by drying. Subsequently, transmission electron microscopy (TEM, Talos F200S, Thermo Fisher Scientific, Waltham, MA, USA) was employed for observation, as depicted in Figure 12. From Figure 12, it can be observed that the ablation product powder of sample CNT0.6CB1.0-P3.0 appears as a fiber–polymer/particle hybrid at the microscopic scale. In previous studies, XRD characterization results have confirmed that laser ablation of PDMS generates amorphous carbon [38]. Preliminary analysis suggests that it comprises amorphous carbon formed from the ablation of added MWCNT, CB, and PDMS. The micrograph in Figure 12 clearly shows fibrous structures resembling MWCNT enveloped by amorphous carbon, with particulate matter adhering to their surfaces.

### 3.3. Analysis of the Synergistic Effect of Piezoresistive Resistance in Laser-Ablated Composite Material Sensors

**Figure 13:**
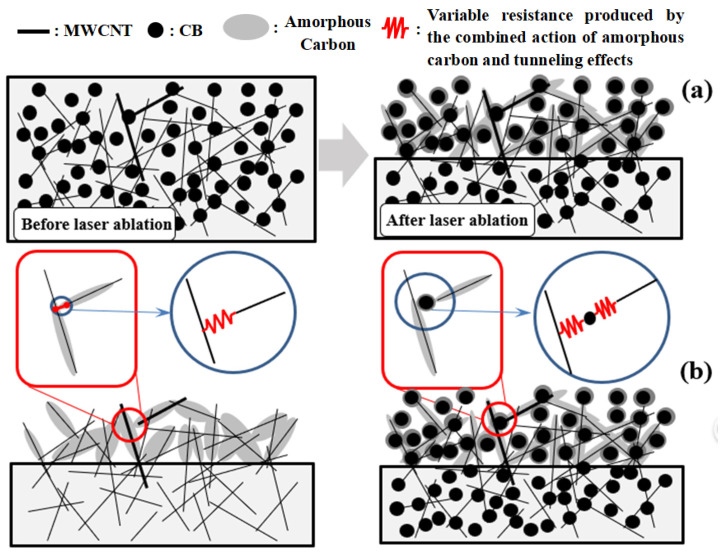
(**a**) Schematic diagram of the internal conductive network structure of MWCNT/CB/PDMS composite before and after laser ablation; (**b**) the comparison of the laser ablation-induced conductive network structure between MWCNT/PDMS composite and MWCNT/CB/PDMS composite.

Due to the high percolation threshold of carbon nano-fillers in PDMS-type rubber materials, the preparation of PDMS-based conductive composites usually requires a large amount of carbon nano-fillers. Laser ablation treatment of nearly insulating PDMS-based composite films with carbon nano-fillers below the percolation threshold can generate “bush-like” conductive layers. As depicted in Figure 13, the laser ablation process induced dual effects on the PDMS matrix: (a) selective removal of polymer components through photothermal decomposition and (b) in situ carbonization of residual PDMS into amorphous carbon layers that conformally coated both CB nanoparticles and MWCNT, ultimately generating a hierarchical conductive network resembling “bush-like” nanostructures.

Previous studies have demonstrated that MWCNT/PDMS composite films develop “bush-like” conductive architectures through laser ablation processes [38]. In contrast, the present work added a very small volume fraction CB particles with low aspect ratio into the conductive structures, which yields a higher proportion of amorphous carbon and enhanced tunneling probability through improved particle proximity. This has a positive significance for improving the piezoresistive sensitivity of the sensor. It is worth noting that due to the fact that the density of MWCNT is much lower than that of CB particles, the volume of MWCNT added in the experiment is far larger than that of CB. When compared in terms of volume fraction, the addition of a trace volume fraction of CB significantly enhances the piezoresistive sensitivity of the sensor.

## 4. Conclusions

This study introduces sensors made of laser-ablated MWCNT/CB/PDMS composite material. The sensors were produced using laser ablation technology, and their piezoresistive performance was evaluated. The experimental results show that at a strain of 5%, the GF value of the sensor test sample CNT0.6CB1.0-P3.0 reaches 584.7, and the sensitivity is much better than that of the traditional carbon nano/polymer homogeneous mixture. Compared with the sensor prepared by ablating a single carbon nanomaterial composite film (MWCNT/PDMS) in the author’s previous study, the sensitivity has increased by approximately 14%. SEM microscopic characterization reveals a “bush-like” conductive structure on the surface of the PDMS-based composite material membrane after laser ablation. TEM images and XRD spectra of the ablation products indicate that the surface of carbon nanomaterials becomes coated with an amorphous carbon layer due to laser ablation. CB, in conjunction with MWCNT forming the primary component of the laser-ablated conductive structure, aids in attaching more amorphous carbon, thereby providing more sensitive variable resistance regions through tunneling effect areas. A minute quantity of CB can act in concert with MWCNTs to synergistically enhance the piezoresistive sensitivity of the sensor. The production process of composite strain sensors offers opportunities to develop high-performance strain gauges and other electronic components. This process demonstrates significant potential in the fabrication of highly sensitive flexible strain sensors.

## Figures and Tables

**Table 1:** Information of various types of composite samples.

Sample	Nanofillers	Addition Amount (wt.)	Sample	Nanofillers	Addition Amount (wt.)
CNT0.2	MWCNT	0.2%	CNT0.6	MWCNT	0.6%
CNT1.0	MWCNT	1.0%	CNT1.2	MWCNT	1.2%
CNT1.5	MWCNT	1.5%	CB0.5	CB	0.5%
CB1.0	CB	1.0%	CB2.0	CB	2.0%
CNT0.6CB0.5	MWCNT/CB	0.6%/0.5%	CNT0.6CB1.0	MWCNT/CB	0.6%/1.0%
CNT0.6CB2.0	MWCNT/CB	0.6%/2.0%	CNT1.0CB0.5	MWCNT/CB	1.0%/0.5%
CNT1.0CB1.0	MWCNT/CB	1.0%/1.0%	CNT1.0CB2.0	MWCNT/CB	1.0%/2.0%

**Table 2:** Information on various types of laser ablation MWCNT/CB/PDMS test samples.

Sample	Laser Power	Sample	Laser Power	Sample	Laser Power
CNT0.2-P1.5	1.5 w	CNT0.2-P3.0	3.0 w	CNT0.2-P6.0	6.0 w
CNT0.6-P1.5	1.5 w	CNT0.6-P3.0	3.0 w	CNT0.6-P6.0	6.0 w
CNT1.0-P1.5	1.5 w	CNT1.0-P3.0	3.0 w	CNT1.0-P6.0	6.0 w
CNT1.2-P1.5	1.5 w	CNT1.2-P3.0	3.0 w	CNT1.2-P6.0	6.0 w
CNT0.2-P9.0	9.0 w	CNT0.6CB0.5-P1.5	1.5 w	CNT1.0CB1.0-P1.5	1.5 w
CNT0.6-P9.0	9.0 w	CNT0.6CB1.0-P1.5	1.5 w	CNT1.0CB2.0-P1.5	1.5 w
CNT1.0-P9.0	9.0 w	CNT0.6CB2.0-P1.5	1.5 w	CNT0.6CB0.5-P3.0	3.0 w
CNT1.2-P9.0	9.0 w	CNT1.0CB0.5-P1.5	1.5 w	CNT0.6CB1.0-P3.0	3.0 w
CNT0.6CB2.0-P3.0	3.0 w	CNT0.6CB0.5-P6.0	6.0 w	CNT1.0CB1.0-P6.0	6.0 w
CNT1.0CB0.5-P3.0	3.0 w	CNT0.6CB1.0-P6.0	6.0 w	CNT1.0CB2.0-P6.0	6.0 w
CNT1.0CB1.0-P3.0	3.0 w	CNT0.6CB2.0-P6.0	6.0 w	CNT0.6CB0.5-P9.0	9.0 w
CNT1.0CB2.0-P3.0	3.0 w	CNT1.0CB0.5-P6.0	6.0 w	CNT0.6CB1.0-P9.0	9.0 w
CNT0.6CB2.0-P9.0	9.0 w	CNT1.0CB1.0-P9.0	9.0 w	CB2.0-P1.5	1.5 w
CNT1.0CB0.5-P9.0	9.0 w	CNT1.0CB2.0-P9.0	9.0 w	CB2.0-P3.0	3.0 w
CB2.0-P6.0	6.0 w	CB2.0-P9.0	9.0 w		

**Table 3:** The resistance of the laser ablation MWCNT/CB/PDMS composite strain sensors.

Sample	Resistance	Sample	Resistance	Sample	Resistance
CB0.5-P1.5	-	CB1.0-P1.5	--	CB2.0-P1.5	-
CB0.5-P3.0	-	CB1.0-P3.0	-	CB2.0-P3.0	-
CB0.5-P6.0	-	CB1.0-P6.0	-	CB2.0-P6.0	32.4 MΩ
CB0.5-P9.0	-	CB1.0-P9.0	-	CB2.0-P9.0	-
CNT0.2-P1.5	-	CNT0.6-P1.5	-	CNT1.0-P1.5	238.1 kΩ
CNT0.2-P3.0	-	CNT0.6-P3.0	-	CNT1.0-P3.0	127.6 kΩ
CNT0.2-P6.0	-	CNT0.6-P6.0	159.6 kΩ	CNT1.0-P6.0	39.6 kΩ
CNT0.2-P9.0	209.3 kΩ	CNT0.6-P9.0	35.1 kΩ	CNT1.0-P9.0	19.7 kΩ
CNT1.2-P1.5	108.3 kΩ	CNT1.2-P3.0	85.1 kΩ	CNT1.2-P6.0	23.8 kΩ
CNT1.2-P9.0	12.3 kΩ				
CNT0.6CB0.5-P1.5	-	CNT0.6CB0.5-P3.0	318.5 kΩ	CNT0.6CB0.5-P6.0	244.7 kΩ
CNT0.6CB0.5-P9.0	109.5 kΩ	CNT0.6CB1.0-P1.5	715.4 kΩ	CNT0.6CB1.0-P3.0	306.6 kΩ
CNT0.6CB1.0-P6.0	218.7 kΩ	CNT0.6CB1.0-P9.0	114.2 kΩ	CNT0.6CB2.0-P1.5	581.7 kΩ
CNT0.6CB2.0-P3.0	404.2 kΩ	CNT0.6CB2.0-P6.0	213.7 kΩ	CNT0.6CB2.0-P9.0	105.5 kΩ
CNT1.0CB0.5-P1.5	203.7kΩ	CNT1.0CB0.5-P3.0	125.4 kΩ	CNT1.0CB0.5-P6.0	35.8 kΩ
CNT1.0CB0.5-P9.0	22.6 kΩ	CNT1.0CB1.0-P1.5	208.1 kΩ	CNT1.0CB1.0-P3.0	114.4 kΩ
CNT1.0CB1.0-P6.0	38.9 kΩ	CNT1.0CB1.0-P9.0	21.7 kΩ	CNT1.0CB2.0-P1.5	188.4 kΩ
CNT1.0CB2.0-P3.0	99.3 kΩ	CNT1.0CB2.0-P6.0	30.6 kΩ	CNT1.0CB2.0-P9.0	19.9 kΩ

**Table 4:** The influence of different CB contents on the initial resistance of thin-film sheets (under the same CNT content and laser power).

CNT Mass Fraction %	Laser Power (W)	CB Mass Fraction %	Initial Resistance (kΩ)
0.6	3	0	Exceed the instrument’s range
0.6	3	0.5	318.5
0.6	3	1	306.6
0.6	3	2	404.2
0.6	6	0	159.6
0.6	6	0.5	244.7
0.6	6	1	218.7
0.6	6	2	213.7
0.6	9	0	35.1
0.6	9	0.5	109.5
0.6	9	1	114.2
0.6	9	2	105.5
1	3	0	127.6
1	3	0.5	125.4
1	3	1	114.4
1	3	2	99.3
1	6	0	39.6
1	6	0.5	35.8
1	6	1	38.9
1	6	2	30.6
1	9	0	19.7
1	9	0.5	22.6
1	9	1	21.7
1	9	2	19.9

## Data Availability

Data are contained within the article.
